# Community Assembly and Stability in the Root Microbiota During Early Plant Development

**DOI:** 10.3389/fmicb.2022.826521

**Published:** 2022-04-21

**Authors:** Kristin Aleklett, Daniel Rosa, Brian John Pickles, Miranda M. Hart

**Affiliations:** ^1^Department of Plant Protection Biology, Swedish University of Agricultural Sciences, Lomm, Sweden; ^2^Department of Biology, University of British Columbia – Okanagan, Kelowna, BC, Canada; ^3^School of Biological Sciences, University of Reading, Health & Life Sciences Building, Whiteknights, United Kingdom

**Keywords:** bacteria, fungi, plant microbiome, priority effects, root microbiota, *Setaria viridis*, soil exposure

## Abstract

Little is known about how community composition in the plant microbiome is affected by events in the life of a plant. For example, when the plant is exposed to soil, microbial communities may be an important factor in root community assembly. We conducted two experiments asking whether the composition of the root microbiota in mature plants could be determined by either the *timing* of root exposure to microbial communities or priority effects by early colonizing microbes. Timing of microbial exposure was manipulated through an inoculation experiment, where plants of different ages were exposed to a common soil inoculum. Priority effects were manipulated by challenging roots with established microbiota with an exogenous microbial community. Results show that even plants with existing microbial root communities were able to acquire new microbial associates, but that timing of soil exposure affected root microbiota composition for both bacterial and fungal communities in mature plants. Plants already colonized were only receptive to colonizers at 1 week post-germination. Our study shows that the timing of soil exposure in the early life stages of a plant is important for the development of the root microbiota in mature plants.

## Introduction

The root microbiota of plants are a key determinant of plant health—yet little is known about the factors that influence its stability. Exposure to microbes during the first weeks of a plant’s life has an important role in shaping the microbiota of mature plants (Green et al., 2006; Barret et al., 2014; Truyens et al., 2014) with consequences for both host productivity and health (Berendsen et al., 2012; Mendes et al., 2013). Understanding the factors responsible for root community assembly is important in order to optimize the microbiome of plants, which is a growing objective of agriculture (Pascale et al., 2020; Trivedi et al., 2020).

There is some evidence that the root microbiota change over the life of an individual plant (Mougel et al., 2006; Houlden et al., 2008; Yu et al., 2012; Chaparro et al., 2014), but studies have also suggested that the community might reach a stable state after the first 2 weeks of development (Ibekwe and Grieve, 2004; Edwards et al., 2015). However, how root communities change during plant development is not well-known (Xiong et al., 2021), nor do we understand how sensitive root communities are to perturbation.

### Window of Opportunity

The ability of microbes to colonize a new root may be controlled by the physiological constraints of the aging root system. For example, physical changes in the root habitat associated with aging could influence which microbes can colonize. In the most broad sense, the reduced proportion of root hairs and increasing amount of hardened surfaces of older roots may create colonization barriers for certain microbial taxa (Watt et al., 2006). This could lead to a diversification of the root habitat in older plants and a root system that be more species-rich, both within and between individuals.

Chemical changes in aging roots, specifically in exudation patterns of carbohydrates and amino acids over time (Badri and Vivanco, 2009; Chaparro et al., 2013), may render the root system more or less attractive to microbial colonizers. For example, root exudates decrease as the plant grows older (Badri and Vivanco, 2009), with visible differences in exudation during the first 2 weeks of growth (Chaparro et al., 2013). In addition, the constituents of the exudates change over time; for instance, phenolic acids, many of which have antimicrobial properties (Cueva et al., 2010), increase after the first weeks of germination (Chaparro et al., 2013). It has also been shown that plants increase their secretion of defense-related proteins such as chitinases, glucanases, myrosinases later in development, specifically in association with flowering (De-la-Peña et al., 2010). Chemical signaling in the root system between plants and microbes may likewise vary throughout development (Lareen et al., 2016) preventing or facilitating microbial colonization (Plett et al., 2011).

It is not known whether these changes constitute a “window of opportunity” for root microbes to colonize the roots, but given the dramatic changes in plant development, plants are unlikely to be equally receptive to microbial colonization throughout their lives, nor throughout their expanding root systems, which represent a diversity of age and size classes in a mature plant. If plants can take up new microbial associations throughout their lives, then we should see a shift in root microbiota of all plant age classes after the introduction of soil. This would lead to a more homogeneous root community among plants, regardless of the age of exposure. Conversely, if there is a “window of opportunity” for root community assembly, then root communities should resist compositional changes even after soil exposure. Thus, mature plants would harbor distinct communities, based on their exposure during the susceptible “window of opportunity.”

### Resistance Hypothesis

Regardless of when plants acquire their root microbiome, they are exposed to different microbial species in their lifetime (Aleklett and Hart, 2013). Microbial propagules disperse naturally through the atmosphere *via* wind-blown dust and rain (Harner et al., 2010; Egan et al., 2014) and by animal vectors including humans (Rosendahl et al., 2009; Lekberg et al., 2011; Nielsen et al., 2016). They are also introduced in the form of bioinoculants or fertilizers (Kokkoris et al., 2019; Rosa et al., 2020). Whether these propagules can establish on a root that has already acquired a root microbial community is unclear, but there is some evidence for this in the phyllosphere (Morella et al., 2020).

It may be that the earliest community to establish may preclude subsequent colonizers (Drake, 1991; Chase, 2003; Debray et al., 2021). Among the earliest would be the seed-dwelling endophytes passed vertically from mother to offspring (Rodríguez et al., 2020; Abdelfattah et al., 2021; Walsh et al., 2021). This so-called priority effect has been documented in microbial communities of both wood-decaying fungi (Fukami et al., 2010) and nectar yeasts (Peay et al., 2012), as well as in mycorrhizal fungi colonizing plant roots (Kennedy et al., 2009; Werner and Kiers, 2015). If so, then a root microbial community could perhaps be “resistant” to colonization by microbes in exogenous soil. If priority effects are not an important determinant of root communities, then a plant when faced with a new species pool of microbes, *via* water, animal, air, or soil movement, would be expected to deviate from the original community. This resistance to colonization may also be related to the *window of opportunity hypothesis*, whereby only plants exposed to new communities during a critical “window” in time would be susceptible to colonization by new microbes.

If priority effects are important for structuring the mature plant microbiome as a whole, then we predict that the original community composition should be more closely aligned with the mature root community, regardless of an influx of new microbial colonizers. In contrast, if plants are susceptible to changes in microbiota post-colonization, then plants exposed to new microbial colonizers should deviate from the original community. In addition, we predict that the root microbiota in plants exposed to microbes from novel microbial communities later in development would be more resistant to change than communities associated with younger plants.

The ability to manipulate the microbiota in plant roots is an area of growing interest, with research beginning to explore the extent to which it is possible to fully exploit the benefits conferred by root symbionts (Ahkami et al., 2017; Pascale et al., 2020; Trivedi et al., 2020). Understanding the assembly processes of the root microbiome is essential if we are to be able to successfully manipulate them.

## Materials and Methods Unique to Each Experiment

The goal of this study was to test whether the timing of exposure to colonizing microbes affects the assembly and development of the mature root microbiota. We tested our hypotheses in two experiments:

### Experiment 1: Window of Opportunity

**Figure 1:**
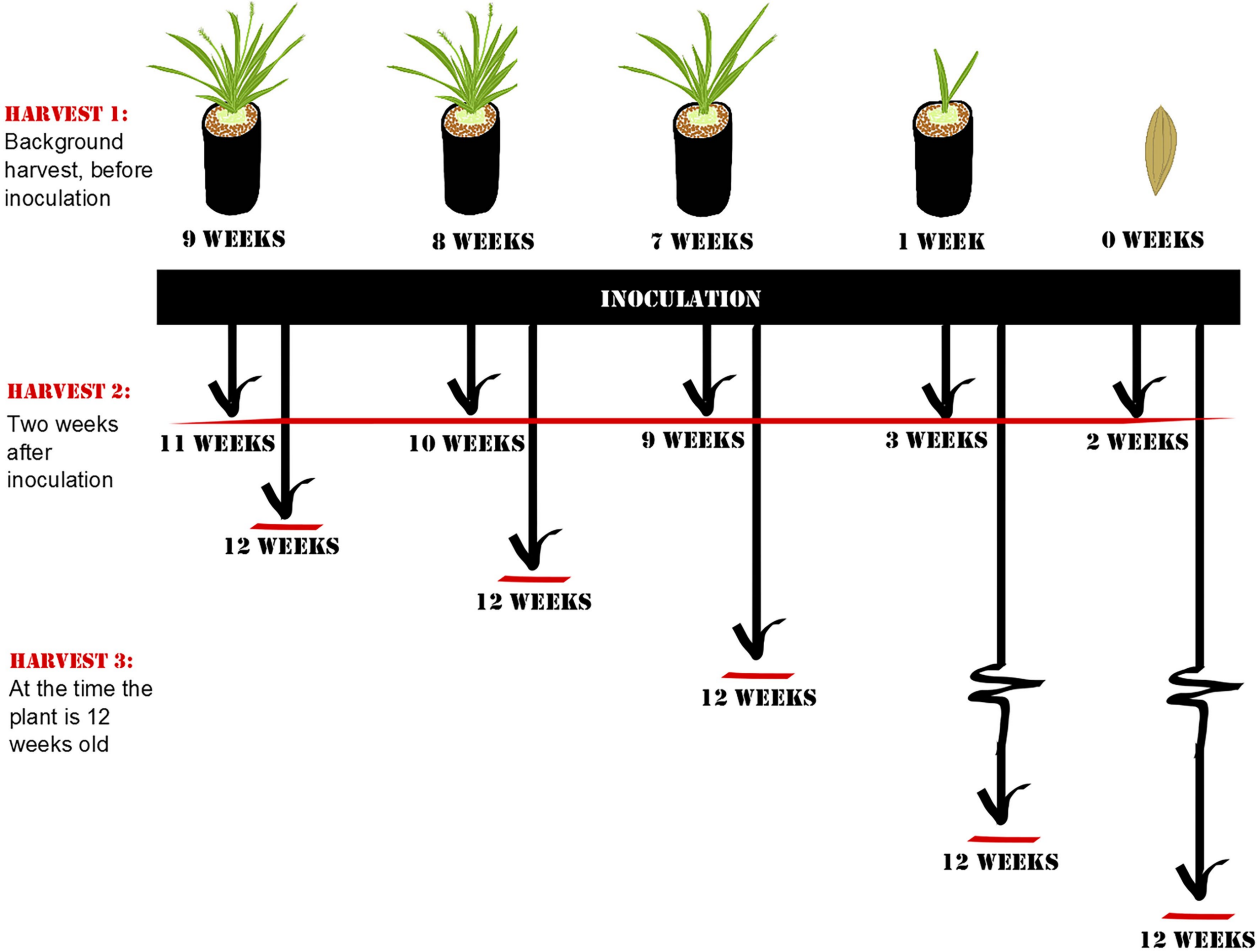
Schematic overview of Experiment 1: Window of opportunity. At each harvest, root samples of *Setaria viridis* were collected. Harvest 1 controls for community composition prior to inoculation and includes plants of representing different developmental stages (flowering, budding, non-reproducing mature plant, seedling, and seed). Harvest 2 was collected 2 weeks after inoculation to control for the length of exposure to the inoculum, and Harvest 3 was collected when the plant was 12 weeks old to control for the age of the plant. The age of the plants at the time of harvest is listed in the diagram.

To test for the age at which plants are most susceptible to root colonization, we introduced soil microbes to plant roots of different developmental stages. Plants were planted at time intervals to create cohorts of different ages in order to be able to apply the soil inoculum at one point in time, avoiding storage effects on the inoculum. Therefore, the only difference among the groups was the age at which they received microbial inoculum (for a more detailed description of the planting and harvesting setup, see Figure 1). At the time of inoculation, plants represented different life stages (seeds, seedlings, adult plants, budding plants, and flowering plants) and ages (0, 1, 7, 8, and 9 weeks old).

Before inoculation, one-third of the plants from all age categories were harvested to determine root microbial communities before inoculation (Harvest 1; for a detailed summary of how many samples were acquired and retained from each harvest, see Supplementary Table S1). The remaining plants were inoculated with a soil-slurry consisting of soil collected from the top 20 cm of rhizosphere soil of a single mature wild specimen of *Setaria viridis* (growing at University of British Columbia – Okanagan campus; 49.939975N, -119.399264W) and autoclaved de-ionized water. Briefly, collected soil was mixed with 250 ml water and sieved through a 2-mm mesh before 1 ml was applied to the rock wool plugs of the plants.

Around 2 weeks after inoculation, half of the remaining plants were harvested to control for the length of exposure to soil (Harvest 2). Roots were collected for DNA extraction (described below). The remaining plants were grown until they reached a total of 12 weeks of age (i.e., 3, 4, 5, 11, or 12 weeks after inoculation; Harvest 3) after which they were harvested and sampled for root communities. By 12 weeks, all plants were mature plants that had not yet started to senesce.

### Experiment 2: Resistance Hypothesis

**Figure 2:**
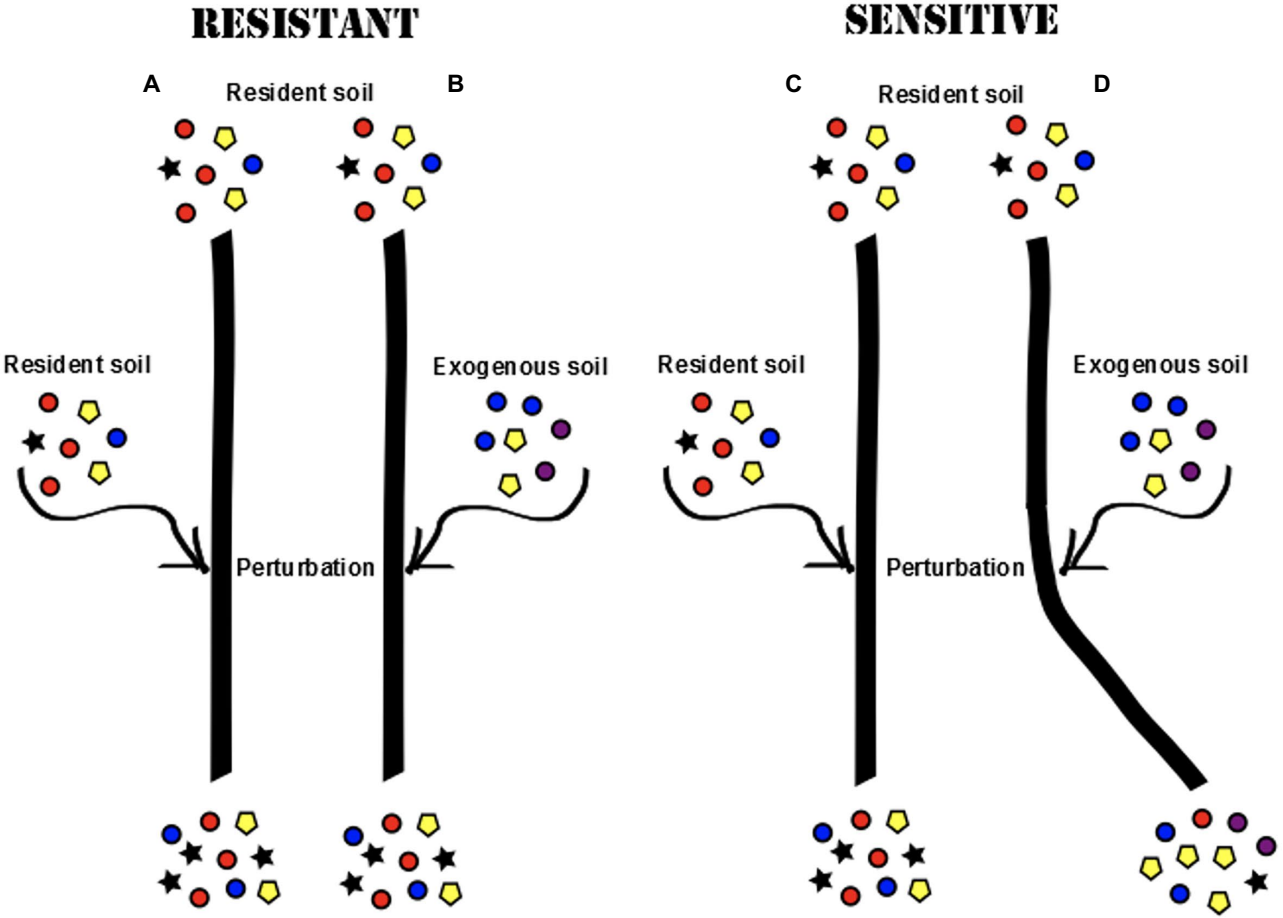
Different root microbial communities may develop in the plant roots if the initial community is resistant or sensitive to perturbation (colors represent different microbial taxa). In a resistant community, the progression of community development would continue in the same direction regardless of exposure to an exogenous soil **(A,B)**. In a sensitive community, the introduction of an exogenous soil would cause a shift in community composition **(C,D)**.

The experiment was set up to study the effect of perturbation, created by the addition of an exogenous soil on resident root microbial communities (for details of the experimental setup, see Figure 2). All pots were initially inoculated with resident soil at the time of planting the seeds. The plants were further introduced to an additional soil perturbation either from the start as seeds (A2), or after one (B2), or 2 weeks (C2) of germinating. These ages were chosen in order to capture differences in early plant development before the root microbiota are thought to reach a stable state [12,13]. In each age class, half of the plants were re-inoculated with the resident soil (A1, B1, and C1) and half of the plants were exposed to a new exogenous soil (A2, B2, and C2).

The two soils used (resident and exogenous) were collected in geographically remote areas with different climates, ecosystems, and plant communities and showed clear differences in properties such as texture, particle size, and soil organic matter content (For more detailed descriptions, see Supplementary Tables S2, S3). Soil inocula were prepared using 250 ml autoclaved de-ionized water and sieved through a 2-mm mesh before being applied to the rock wool plugs of the plants. Each plant received a total of 2.5 ml soil slurry at the time of planting, and an additional 2.5 ml slurry at the time of perturbation.

To control for the length of exposure and let the perturbation take effect, all plants were grown for an additional 3 weeks post-perturbation before harvest, creating three different age classes among the harvested plants (Harvest 1: 3 weeks old, Harvest 2: 4 weeks old, and Harvest 3: 5 weeks old). In order to assess the effect of type of perturbation (resident or exogenous), root communities harvested from plants of the same age class were compared, and root communities in plants only exposed to the resident soil (A1, B1, and B2) were used as a baseline for expected community development. Among plants that were exposed to the exogenous soil (A2, B2, and C2), plants in which we saw a divergence in microbial community development were classified as sensitive to invasion, whereas plants hosting communities that continued the same community development despite the introduction of an exogenous soil were classified as resistant. This allowed us to examine questions such as: are we able to change the direction of community development in the root microbiota by introducing an exogenous soil to the system and is the root microbiota more or less resistant to invasion at different ages during early development?

Roots were harvested from seven plants of each treatment (A1, A2, B1, B2, C1, and C2), with a total of 14 plants per harvest. All roots were collected from inside of the rock wool plug in order to control for differences in the immediate environment surrounding the roots. Roots were further rinsed with autoclaved de-ionized water and dried on a sterile filter paper. A subsample of 0.10 g (wet weight) from the root system (representing a collection of roots of different ages) was collected from each plant, which was then used for DNA extraction.

## Materials and Methods Common to Both Experiments

### Host and Growing Conditions

For both studies, we used *Setaria viridis* (L.) P. Beauv. as a host as it has a short lifecycle (Kennedy et al., 2009; Kokkoris et al., 2019), making it an ideal host plant for this study. Clonal seeds of *S. viridis* (accession A 10.1) were donated by the Brutnell Lab at the Boyce Thompson Institute for Plant Research, New York, United States. Seeds were sown in Sure Roots plug trays (TO38SR, Stuewe and Sons, INC.) containing purely artificial materials to replace soil [rock wool plugs (Grodan A-OK 1.5 inch starter plugs), surrounded by Turface (Turface, MVP)]. Plants were then grown in a growth chamber (Conviron CMP6010) with 12-h/12-h (day/night) photoperiod, 28/22°C (day/night). Watering was performed daily with autoclaved de-ionized water, and fertilization occurred once per week with 2.5 ml Technigro 17-5-24 plus (1:200 dilution).

### DNA Extraction and Amplification

For both studies, whole plants were destructively harvested, and roots were rinsed with autoclaved, de-ionized water, and dried with sterile filter paper. A subset of 0.1 g root tissue (wet weight) was sampled from each plant from inside the rock wool plug (controlling for immediate root environment), representing a subset of roots of various ages. Roots were frozen with liquid nitrogen and then manually crushed using a sterile pestle. Attempts were made to extract DNA from the seeds used in the experiment (both as individual seeds and as a mix of 10 seeds), but without successful amplification of either bacteria or fungi. Samples were kept at −80°C until DNA extraction (DNeasy plant mini kit; QIAGEN) according to the manufacturer’s protocols.

#### Bacteria

The V5 and V6 regions of the 16S SSU rRNA gene were amplified to characterize the bacterial community, using the forward primer 799f (Chelius and Triplett, 2001) and the reverse “universal” bacterial primer 1115r (Reysenbach and Pace, 1995). Each sample was also labeled with a unique 10 base pair (bp) identification barcode associated with the forward primer, as well as a 4-bp TCAG key, and a 21-bp adapter for 454 sequencing. The forward primer used in our study (799f) was designed to exclude chloroplast DNA and give a mitochondrial product approximately 1.5 times the size of the bacterial product (Kõljalg et al., 2013). Each PCR consisted of 25 μl (14.85 μl H_2_O, 5 μl GoTaq Flexi buffer, 1 μl BSA, 2 μl MgCl_2_, 0.5 μl dNTP, 0.2 μl forward primer, 0.2 μl reverse primer, 0.25 μl GoTaq Flexi, and 1 μl DNA template). Samples were initially denatured at 95°C for 5 min and then amplified by using 30 cycles of 95°C for 1 min, 61°C for 1 min, and 72°C for 1 min with a final extension of 7 min at 72°C.

#### Fungi

The ITS2 region was amplified for fungal identification using the primers fITS7 (Kuczynski et al., 2010) and ITS4 (Lareen et al., 2016). Each sample was also labeled with a unique 10 base pair identification barcode associated with the forward primer, as well as a 4-bp TCAG key, and a 21-bp adapter for 454 sequencing. PCR was carried out where each sample consisted of 25 μl (12.75 μl H_2_O, 5 μl GoTaq Flexi buffer, 1 μl BSA, 3.5 μl MgCl_2_, 0.5 μl dNTP, 0.5 μl forward primer, 0.5 μl reverse primer, 0.25 μl GoTaq Flexi, and 1 μl DNA template). Samples were initially denatured at 94°C for 5 min and then amplified by using 34 cycles of 94°C for 45 s, 61°C for 45 s, and 72°C for 1 min. A final extension of 7 min at 72°C was added at the end of the program to ensure complete amplification of the target region.

All samples were amplified in triplicate. Negative controls (no-template) were included in all steps of the process to check for primer or sample DNA contamination. Samples were sent to the Laboratory for Advanced Genome Analysis (LAGA) at the Vancouver Prostate Centre (University of British Columbia, Vancouver) for purification and 454 sequencing using the GS-FLX Titanium sequencing platform, emulsion PCR and Lib-L chemistry for unidirectional sequencing (Roche, Branford, CT, United States).

While Illumina is known to produce more sequences for analysis, pyrosequencing data still provide a valid representation of microbial community composition. In fact, for bacteria, it has been shown that analysis of as few as 100 sequences of a bacterial community will still produce the same results in terms of detecting differences between samples and treatments (Kuczynski et al., 2010). For fungi, the suggested limit for how many sequences are needed to properly depict differences in community composition is thought to be a bit higher, around 400 sequences/sample, but tests analyzing the same samples with 454 and Illumina techniques have still generated the same results in terms of differences in α- and β-diversity (Smith and Peay, 2014).

### Sequence Analysis and Statistics

Raw sequences and supporting information are available at: https://doi.org/10.6084/m9.figshare.19291934.

Sequence data were processed through the QIIME pipeline (Caporaso et al., 2010a), where sequences were clustered using the uclust (Edgar, 2010; bacteria) or mothur (Schloss et al., 2009; fungi) algorithm to pick Operational Taxonomic Units (OTUs), which were defined at 97% sequence similarity. Bacterial sequences were further aligned against the Greengenes database (13_8 database) with PyNast (DeSantis et al., 2006; Caporaso et al., 2010b) using the rdp classifier (Wang et al., 2007), and chimeric sequences were removed with ChimeraSlayer (Haas et al., 2011). Fungal sequences were identified using the UNITE (12_11_otus database; Abarenkov et al., 2010; Kõljalg et al., 2013) and the rdp classifier (Wang et al., 2007).

A total of 347,813 bacterial and 353,151 fungal raw sequences were captured in the samples of the Window of Opportunity study. Among those, 1,442 bacterial and 961 fungal OTUs were found. In the Resistance Hypothesis study, we recorded 129,680 bacterial and 115,424 fungal raw sequences, representing 1,578 and 1,024 OTUs, respectively. To adjust for unequal numbers of sequences among samples, the Window of Opportunity bacterial dataset was rarefied at 2,000 sequences/sample and the fungal data set was rarefied at 1,000 sequences/sample before further analysis of α- and ß-diversity. For the Resistance Hypothesis experiment, data were rarefied at 1,648 (bacteria) and 1,267 (fungi) sequences per sample in order to adjust of unequal sampling. This allowed us to retain all samples for further analysis (Supplementary Table S1; Supplementary Figures S5, S6).

Rarefying reduced the number of samples available for analysis (Supplementary Table S1). We confirmed the results of our ß-diversity PERMANOVA analysis by performing the same statistical procedures on non-rarefied data that was independently filtered and transformed using the regularized log (rlog) transformation on the log2 scale using DESeq2 (Love et al., 2014; McMurdie and Holmes, 2014). The rlog-transformation is recommended if sequence depth varies widely between samples (Love et al., 2014).

Operational Taxonomic Unit richness (α-diversity) was analyzed for both bacterial and fungal data using the “phyloseq” (McMurdie and Holmes, 2013) and “vegan” (Oksanen et al., 2013) packages in R. Observed OTU richness (Observed) in each sample was calculated and compared between harvests and inoculation treatments using *t*-tests or a Wilcoxon test (depending on whether the data were normally distributed), and ANOVAs (Type III SS). Singletons were retained for analysis of α-diversity but removed from the data set before analysis of ß-diversity. Generally, it is recommended that singletons are retained in richness estimation, as many estimators use singletons and doubletons to model species accumulation (Chao et al., 2005). However, due to concerns that next-generation sequencing of fungi produces many singletons that might be largely artefactual (Tedersoo et al., 2010; Smith and Peay, 2014), we confirmed that the results obtained displayed the same general pattern with singletons removed.

Dissimilarities in bacterial and fungal communities between samples were calculated using the Bray–Curtis measure (Bray and Curtis, 1957) for the rarefied dataset and Euclidian distances for the rlog-transformed dataset. As the rarefied datasets are comprised of integers, they are transformed into dissimilarity matrices. The transformed datasets are continuous variables, which have already been converted into a matrix of numerical values that preserve the patterns of variance within the dataset. Thus, for the rarefied data, we constructed distance matrices using ß-diversity metrics, and for the transformed data, we constructed distance matrices using Euclidean distance metrics.

We tested for significant differences in ß-diversity between treatments using PERMANOVA (Type III SS; 9,999 permutations) to account for unequal sample sizes (Anderson, 2005). Pairwise permutational comparisons between treatments performed for samples. Scatter plots (2-D) of principal coordinates analysis (PCoA) were generated in PRIMER-E (Clarke and Gorley, 2006) and used to visualize the greatest amount of variability in the pairwise dissimilarities between samples.

Significant differences in Bray–Curtis dissimilarities between clusters observed in the PCoAs were further compared by a similarity percentage analysis (SIMPER) at the order level (using PRIMER-E), identifying which bacterial and fungal orders contributed most to the variation seen between samples.

## Results

### Experiment 1: Window of Opportunity

#### Effect of Inoculation on Root Microbiota (Harvest 1 Compared to Harvest 2)

##### Bacteria

**Figure 3:**
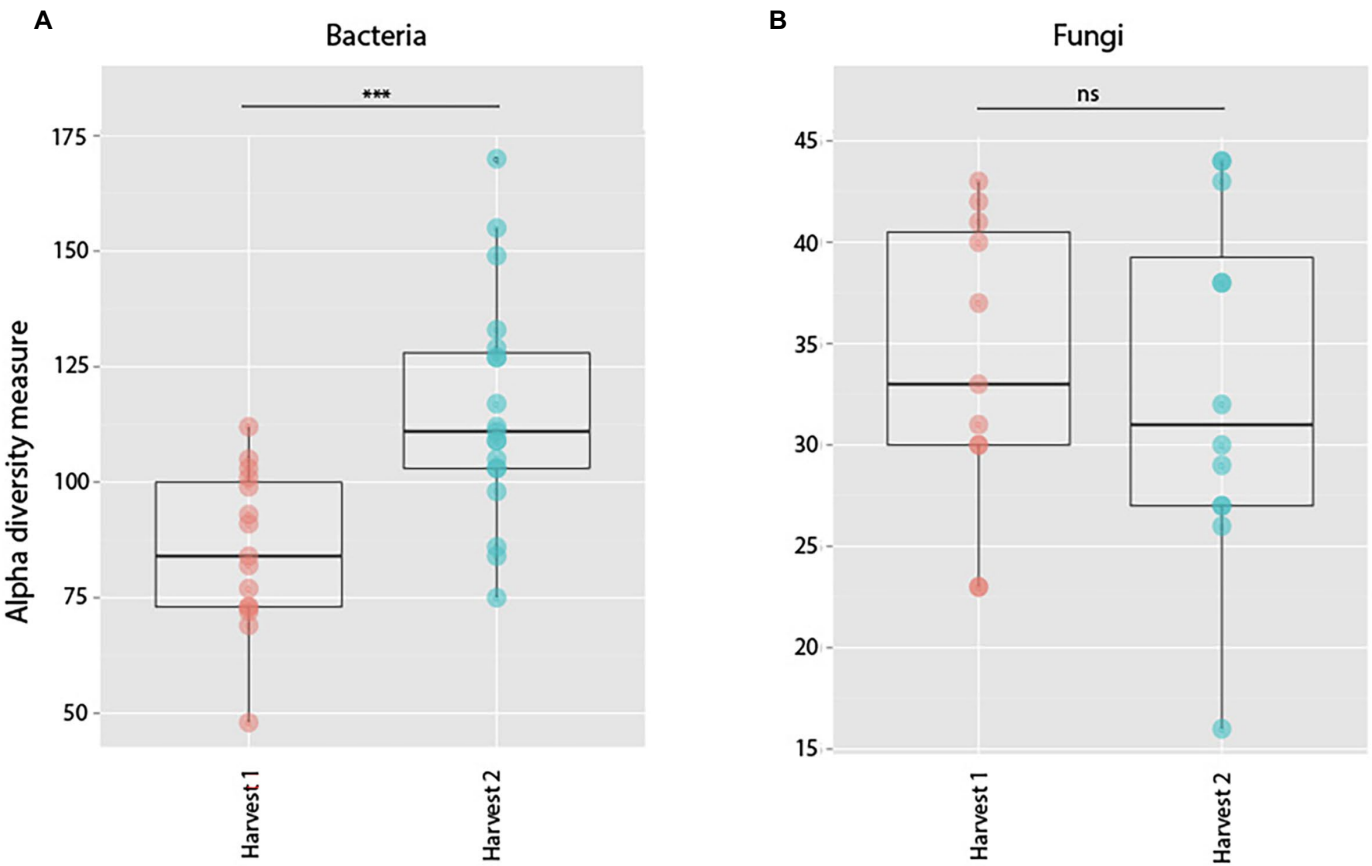
Alpha diversity measures of observed species richness in bacterial **(A)** and fungal **(B)** communities in roots harvested prior to (Harvest 1), or 2 weeks after (Harvest 2) exposure to soil. Each dot represents a sample. Soil exposure to an un-colonized root led to a significant increase in bacterial (*t* = −4.29, *p* = 0.0001) but not fungal (*t* = 1.15, *p* = 0.26) richness. ***indicates *p* < 0.001.

**Figure 4:**
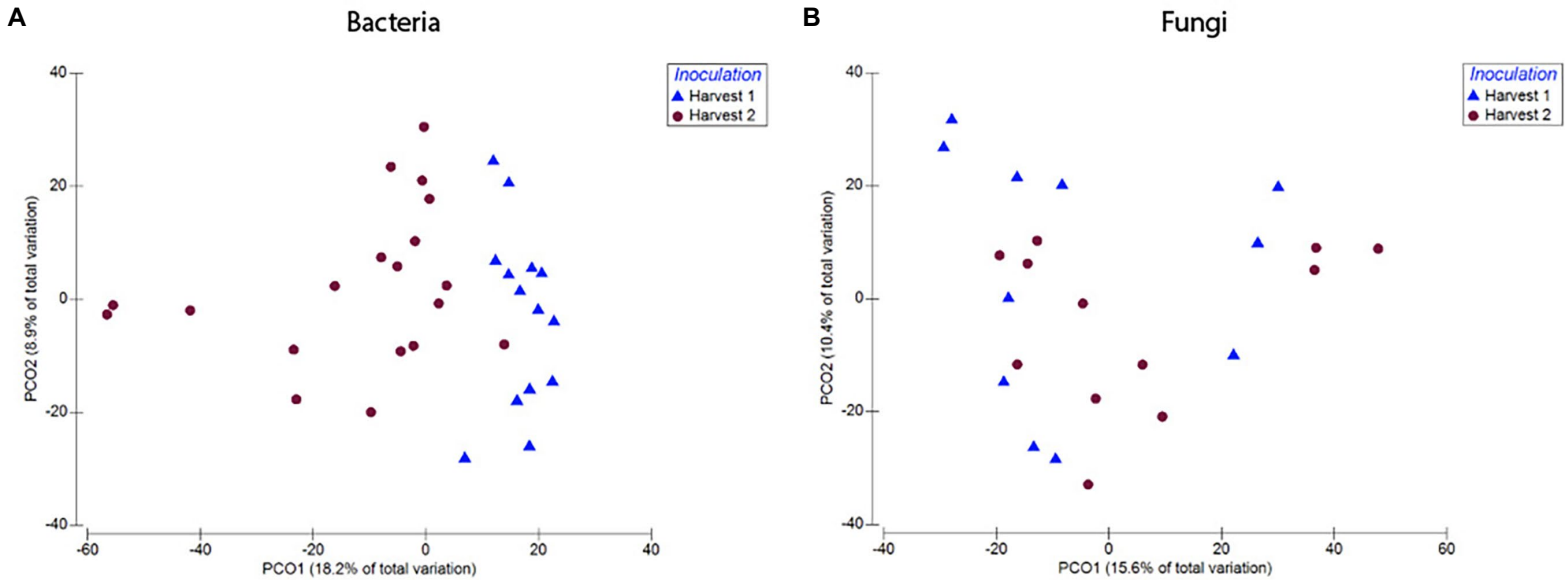
Compositional differences in bacterial **(A)** and fungal **(B)** communities of roots harvested immediately prior to, and 2 weeks after, exposure to soil. Bacterial communities differed significantly between roots harvested before and after inoculation (pseudo-*F* = 2.89, *p* = 0.0001). There was also a difference between fungal communities in roots harvested before and after inoculation (pseudo-*F* = 1.57, *p* = 0.03). Dots represent pairwise differences (measured as Bray–Curtis distances).

Bacterial root communities changed significantly for plants of all ages when introduced to the soil inoculum. Even though a “background” community was already established at the time of inoculation (Harvest 1), exposure to the soil slurry increased OTU richness for all age classes, regardless of whether the data were examined with (*t* = −4.29, *p* = 0.0001) or without (*t* = −4.76, *p* = <0.0001) singletons included (Figure 3), and changed the microbial community composition of the roots (pseudo-*F* = 2.89, *p* = 0.0001; Figure 4). This was observed when comparing samples harvested before inoculation (Harvest 1) to samples harvested 2 weeks after inoculation (Harvest 2). Analysis of the non-rarefied dataset confirmed the statistical difference between bacterial communities before and after inoculation (pseudo-*F* = 2.13, *p* = 0.0001; Supplementary Table S4).

**Figure 5:**
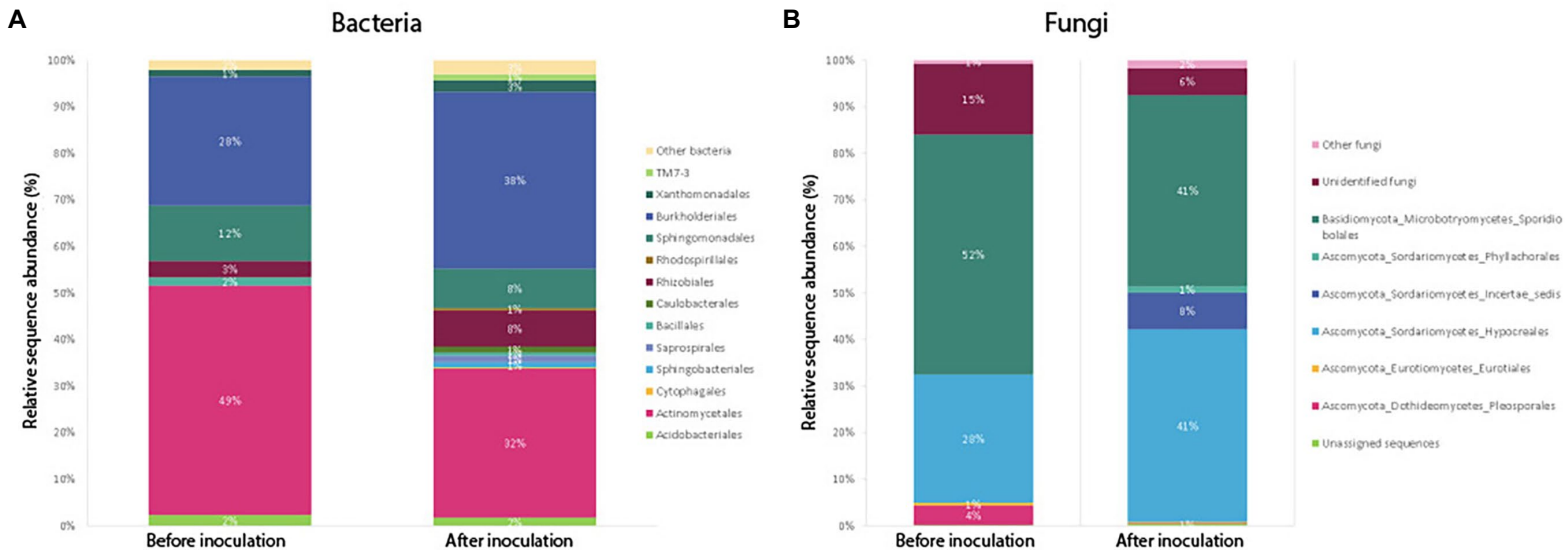
Average relative abundance of sequences belonging to bacterial **(A)** and fungal **(B)** orders, compared between samples harvested before (Harvest 1) and after inoculation (Harvest 2). For Harvest 1 and Harvest 2, the bars show an average community composition based on a combination of all samples from that harvest. Sequences were classified to the level of orders, and orders representing less than 1% of the community have been grouped as “Other.”

A closer look at the community composition before and after inoculation showed that the most dominant bacterial order in un-inoculated roots was Actinomycetales (Figure 5), making up as much as 49% of the average community (average relative abundance) sampled across plants of different ages. Once plants were inoculated with the soil slurry, Burkholderiales became more prominent in the root system. In plant roots harvested 2 weeks after inoculation, Burkholderiales made up 38% of the total community (Figure 5). Rhizobiales also increased post-inoculation from 4 to 8% of the average bacterial community. Sphingobacteriales, Saprospirales, Caulobacterales, and Enterobacteriales also increased from 0 to 1% of the average bacterial community post-inoculation. The same trends of increasing amounts of Burkholderiales and Rhizobiales in the communities post-inoculation were also observed when we compared only plants harvested at the same age (9 weeks) before and after inoculation (Supplementary Figure S1).

Results from the SIMPER analysis showed that overall, differences between samples harvested before and after inoculation were mainly driven by differences in the average relative abundance of the orders Actinomycetales (31.65%), Burkholderiales (25.62%), Sphingomonadales (14.26%), Rhizobiales (6.25%), and Acidobacteriales (5.23%) which showed the highest percentage contribution (listed in brackets) to the average dissimilarity between groups.

##### Fungi

Soil inoculation had no significant effect on fungal community richness regardless of whether the data were analyzed with (*t* = 1.15, *p* = 0.26) or without (*W* = 138, *p* = 0.21) inclusion of singletons (Figure 3), but altered the composition of the overall community significantly (Figure 4; pseudo-*F* = 1.57, *p* = 0.03). However, this compositional effect was not significant at the *α* = 0.05 level when considering the non-rarefied dataset (pseudo-*F* = 1.37, *p* = 0.06; Supplementary Table S4).

Compositionally, comparing the average fungal community in samples before and after inoculation, the biggest change was seen in the order Sporidibolales, which made up 43% of the community in un-inoculated plants, but only 28% post-inoculation (Figure 5). In addition, an unknown order of Dothideomycetes increased from 0 to 6% post-inoculation, as well as ones belonging to an unknown order of Sordariomycetes (from 0 to 4%). When we compared only plants harvested at the same age (9 weeks) before and after inoculation, we saw that the amount of fungi belonging to the order Hypocreales increased post-inoculation from 28 to 41% on average, whereas the proportion of Sporidiobolales and unidentified fungi decreased (Supplementary Figure S1).

Results from the SIMPER analysis showed that overall, differences between samples harvested before and after inoculation were mainly driven by the orders Sporidiobolales (28.9%), Hypocreales (28.1%), Unidentified fungi (16.7%), and Eurotiales (7.8%), which had the highest percentage contribution to the average dissimilarity between Harvest 1 and Harvest 2.

#### Effect of Exposure Time on Root Microbiota (Harvest 2)

##### Bacteria

Plants inoculated at different ages but exposed to the inoculum for the same length of time (Harvest 2) showed a significant difference in observed bacterial OTU richness (*F* = 5.9, *p* = 0.005), as well as a significant difference in community composition between plants inoculated at different ages (pseudo-*F* = 2.22, *p* = 0.0002). This trend was also confirmed in the analysis of the non-rarefied dataset (pseudo-*F* = 2.26, *p* = 0.0001; Supplementary Table S4). Pairwise comparisons of the Bray–Curtis dissimilarities further revealed that the significant difference was primarily between plants inoculated at ages of 0 and 1 week as compared to 7 (*p* = 0.018), 8 (*p* = 0.019), and 9 weeks (*p* = 0.028), whereas there was no significant difference between 0 weeks and 1 week or between 7, 8, and 9 weeks.

##### Fungi

Only plants inoculated at 7, 8, and 9 weeks were retained in the analysis of fungal communities, as we were unable to successfully amplify fungi from the plants inoculated at weeks 0 and 1. Further, only plants inoculated at 8 and 9 weeks contained enough samples to be statistically compared post-rarefaction. There was no significant difference in observed OTU richness (*t* = 0.82, *p* = 0.43) between inoculation at week 8 or 9. There was also no significant difference in fungal community composition between these samples based on a pairwise comparison of Bray–Curtis dissimilarities (*t* = 0.99, *p* = 0.46), or when the data were analyzed without rarefaction including more samples (Supplementary Table S4; pseudo-*F* = 0.97, *p* = 0.59).

#### Effect of Plant Age on Root Microbiota (Harvest 3)

##### Bacteria

**Figure 6:**
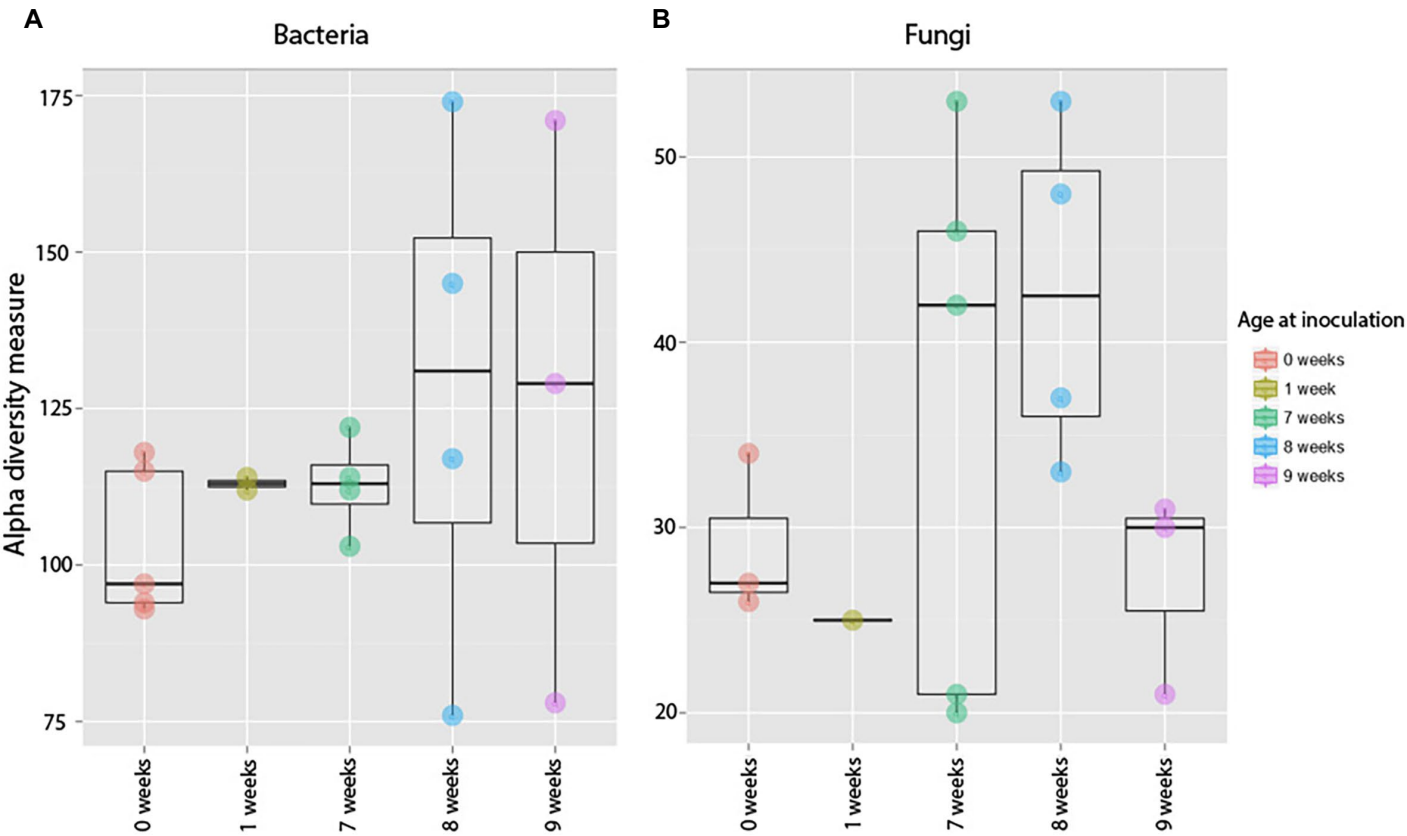
Alpha diversity measures of bacterial **(A)** and fungal **(B)** communities in 12-week-old roots that were exposed to soil at different developmental stages. Each dot represents a sample, and the variation among samples is calculated using observed Operational Taxonomic Unit (OTU) richness. Timing of exposure had no effect of the community composition of mature plants (*F* = 0.66, *p* = 0.59) or fungal (*F* = 1.55, p = 0.26).

**Figure 7:**
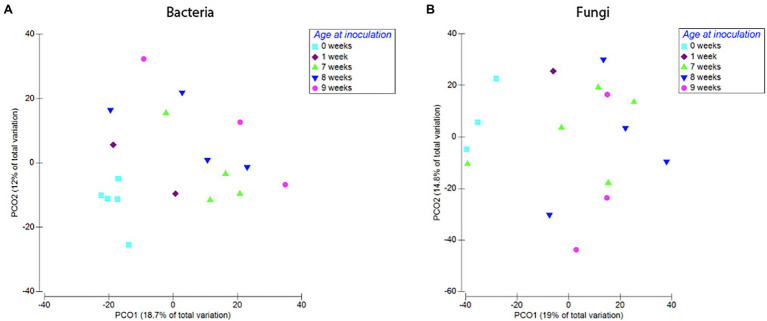
Compositional differences in bacterial **(A)** and fungal **(B)** communities of roots harvested at 12 weeks. Plants inoculated at different developmental stages hosted distinct bacterial (Pseudo-*F* = 1.45, *p* = 0.008) and fungal communities (pseudo-*F* = 1.38, *p* = 0.03). Dots represent pairwise differences (measured as Bray–Curtis distances).

We found no difference in OTU richness between plants inoculated at different ages and harvested at maturity (Harvest 3; *F* = 0.66, *p* = 0.59; Figure 6), but there was a significant difference in community composition (pseudo-*F* = 1.45, *p* = 0.008; Figure 6). This compositional difference was confirmed by the analysis of the non-rarefied dataset (pseudo-*F* = 1.29, *p* = 0.02; Supplementary Table S4). In general, plants inoculated closer in age hosted bacterial communities that were more similar to each other (Figure 7), and pairwise comparisons showed that this trend was driven primarily by the difference between plants inoculated as seeds (0 weeks) vs. those inoculated at 7 weeks (*p* = 0.007), 8 weeks (*p* = 0.008), and 9 weeks (*p* = 0.01). A difference between age-groups was also seen in survival rates post-inoculation, as a majority of plants inoculated at 1 week did not survive to maturity (Supplementary Table S5).

Compositionally, we could see a trend of plants exposed to soil later in life (at 7, 8, or 9 weeks old) hosting bacterial communities with a higher proportion of bacteria belonging to the order Actinomycetales (18, 45, and 21%) and a lower proportion of the order Rhizobiales (11, 4, and 5%) when compared to plants inoculated as seeds or 1-week-old seedlings (Supplementary Figure S2).

##### Fungi

Timing of soil exposure had a less pronounced effect on fungi compared to bacterial communities. Like bacteria, there was no difference among fungal OTU richness for plants inoculated at different developmental stages but harvested at the same age (Harvest 3; *F* = 1.55, *p* = 0.26; Figure 6). However, a comparison of ß-diversity using Bray–Curtis dissimilarities between communities in roots inoculated at different developmental stages, but harvested at 12 weeks (Harvest 3) showed that fungal communities were compositionally different (pseudo-*F* = 1.38, *p* = 0.03; Figure 7; Supplementary Table S4). Pairwise comparisons of plants inoculated at different ages showed that this was primarily driven by differences between samples inoculated at week 0 and those inoculated at week 7 (*p* = 0.10), week 8 (*p* = 0.05), and week 9 (p = 0.10). Analysis of the non-rarefied dataset showed a similar difference between root systems inoculated at different developmental stages (pseudo-*F* = 1.17, *p* = 0.06; Supplementary Table S4).

When fungal community composition was compared at the order level in plants harvested at 12 weeks old (Harvest 3), plants inoculated as seeds (0 weeks) stood out as different from the other age categories, as their roots were extremely dominated by Xylariales, an order that was not dominant in samples from the two first harvests (Supplementary Figure S2).

### Experiment 2: Resistance Hypothesis

#### Effect of Novel Community on Root Microbiota

##### Bacteria

**Figure 8:**
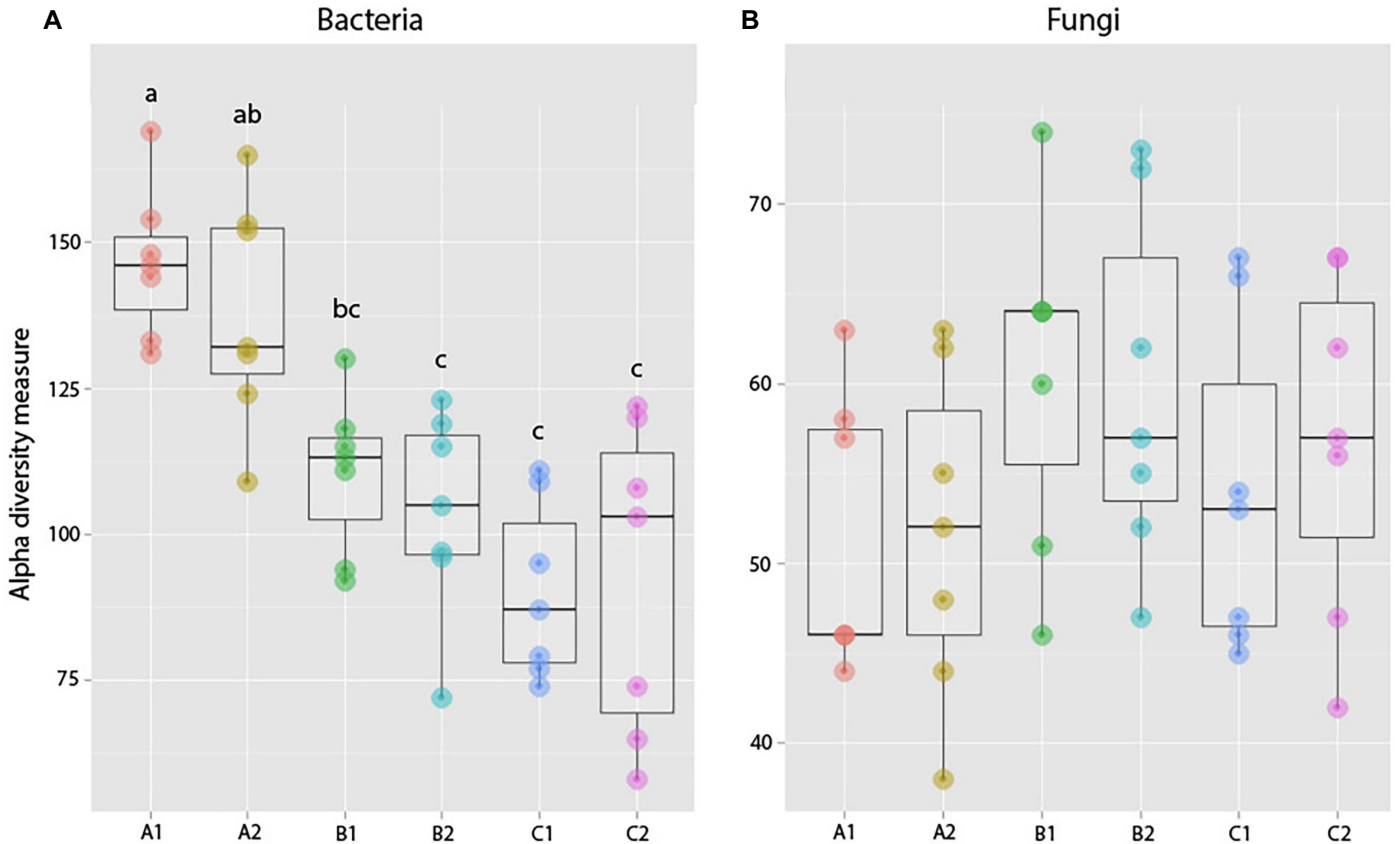
Observed species richness of bacterial **(A)** and fungal **(B)** root communities among roots exposed to resident (1) or exogenous (2) soils at different ages (a, b, and c). There was an overall decrease in bacterial richness over the course of the experiment, but no significant difference in richness between plants of the same age but with different soil exposure. For fungal communities, results show no significant difference between plants with different soil exposures. These observations were in accordance with Duncan test results (significant differences indicated by different lowercase letters).

Pairwise comparisons (Duncan test) between samples in the same age class but exposed to the resident or exogenous soil showed no significant difference in bacterial richness (A1–A2: *p* = 0.95, B1–B2: *p* = 0.98, and C1–C2: *p* = 0.10) between treatments for plants exposed to microbes from novel microbial communities at any age (Figure 8).

PERMANOVA results showed that for bacterial communities, the two factors Harvest (comparing between harvest 1, 2, and 3) and Exposure (comparing between plants exposed to either only the resident or a combination of the resident and the exogenous soil) both had a significant effect on community composition (based on Bray–Cutis dissimilarities). However, there was no significant interaction between the two factors (Supplementary Table S6).

**Figure 9:**
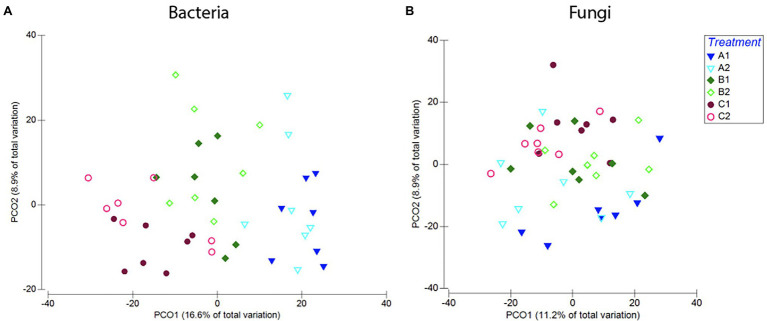
Compositional differences in bacterial **(A)** and fungal **(B)** communities of roots across age classes [seeds (a), 1-week-old seedlings (b), or 2-weeks-old seedlings (c)] and exposure to either resident soil (1) or exogenous soil (2). For bacteria, root communities differed among plants harvested at different ages, but there was no clear distinction between plants with different soil exposure harvested at the same age. For fungi, there is no clear separation between samples with different soil exposure of any age. These trends were confirmed in PERMANOVA comparisons of the Bray–Curtis dissimilarities (Supplementary Table S6).

Further pairwise PERMANOVA comparisons of differences in community composition (Bray–Curtis dissimilarities) between plants harvested at the same age showed a significant difference between plants with different soil exposure when comparing plants that were exposed to microbes from novel microbial communities at 1 week old (treatment B1 and B2; *p* = 0.007, *t* = 1.28; Supplementary Table S6; Figure 9). However, plants exposed to microbes from novel microbial communities either from start (A1, A2) or after 2 weeks of germination (C1, C2) did not show a significant divergence in bacterial community composition (A1–A2: *p* = 0.1, *t* = 1.12; C1–C2: *p* = 0.35, *t* = 1.02; Supplementary Table S6; Figure 9).

Looking closer at community composition, we could see that overall, plants exposed to the exogenous soil (A2, B2, and C2) hosted a larger proportion of bacteria belonging to the order Enterobacteriales, and a smaller proportion of the orders Xanthomonadales, Sphingomonadales, and Bacillales (Supplementary Figure S3). SIMPER analysis showed that the orders Actinomycetales (23%), Burkholderiales (18%), Xanthomonadales (16%), Enterobacteriales (15%), and Sphingomonadales (11%) contributed to 83% of the variation seen between plants with different soil exposures. In samples exposed to microbes from novel microbial communities with the exogenous soil at 1 week old (A2), we saw an on average larger proportion of Burkholderiales (27%), compared to plants of the same age only exposed to the resident soil (A1; 19%; Supplementary Figure S3).

##### Fungi

Pairwise comparisons showed no significant difference in fungal community richness between plants harvested at the same age but with different soil exposure (A1–A2: *p* = 0.99, B1–B2: *p* = 0.99, and C1–C2: *p* = 0.99; Figure 8). PERMANOVA results comparing community composition through Bray–Curtis dissimilarities showed a significant effect of Harvest but not soil exposure on community composition, and there was also no significant interaction between the two factors examined. Pairwise comparisons of treatments further confirmed these results (A1–A2: *p* = 0.19, *t* = 1.10; B1–B2: *p* = 0.86, *t* = 0.88; and C1–C2: *p* = 0.28, *t* = 1.05; Figure 9; Supplementary Table S6) as there was no significant difference between root communities in plants with different soil exposure exposed to microbes from novel microbial communities at any age examined.

Examining the fungal community composition (Supplementary Figure S4), it was clear that the order Hypocreales was dominating the community throughout the experiment and across all treatments. When we examined the community at a higher taxonomic resolution, we found that this dominance was created by a high presence of the genus *Fusarium*.

#### Effect of Plant Age and Introduction of Novel Community on Root Microbiota

##### Bacteria

**Figure 10:**
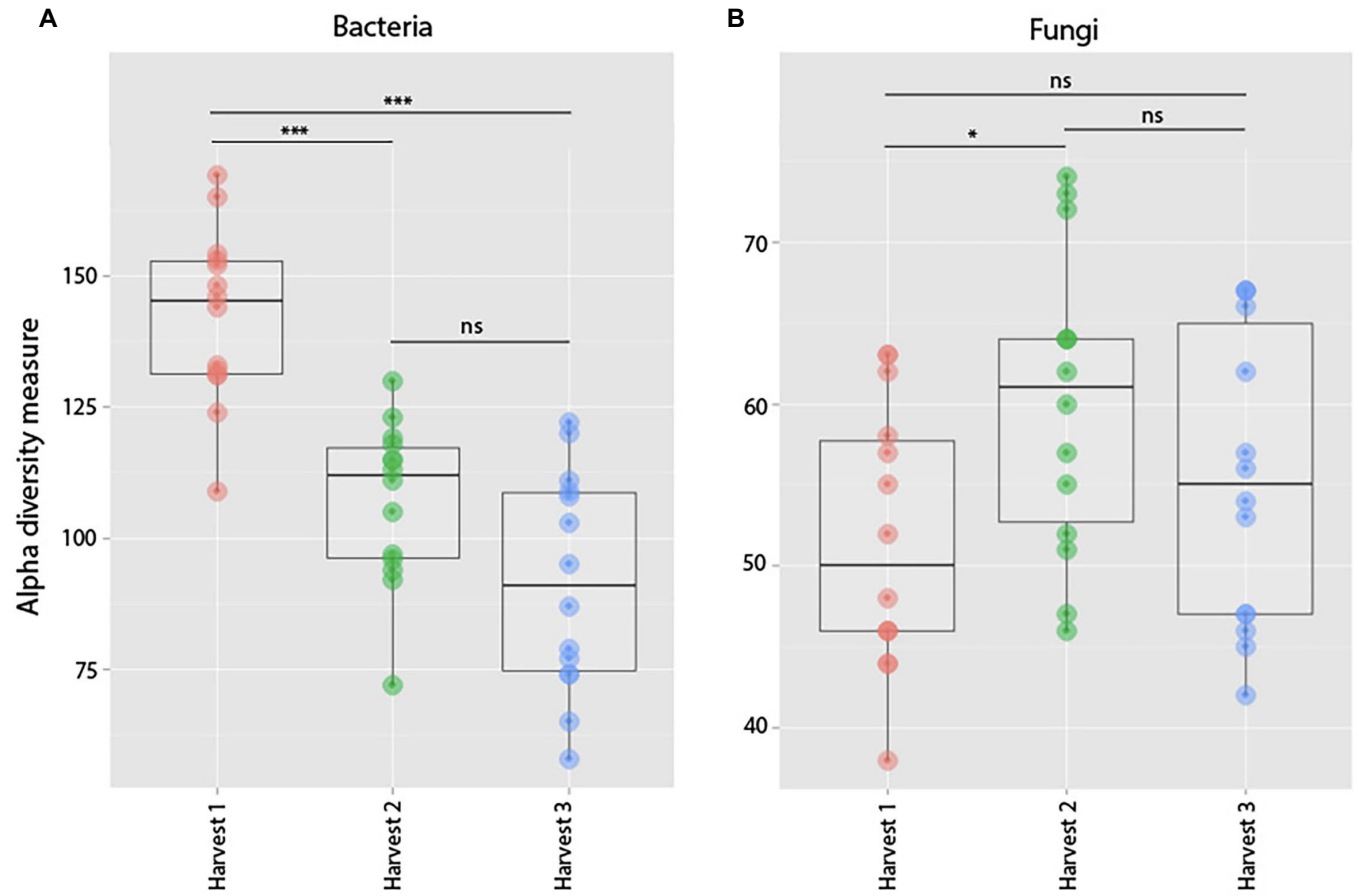
Observed species richness of bacterial **(A)** and fungal **(B)** root communities among harvests. (Harvest 1 = 4 weeks. Harvest 2 = 5 weeks, and Harvest 3 = 12 weeks) that were exposed to resident soil exogenous soil. Bacterial richness decreased significantly between Harvests 1 and 2. For fungi, there was no significant change in richness between harvests. ***indicates *p* < 0.001.

Comparing bacterial richness among harvests, there was a clear trend of plants harvested at an older age (Harvest 2 and Harvest 3) hosting bacterial communities with lower species richness than plants harvested at 3 weeks old (Harvest 1; *p* = <0.0001, *F* = 29.97; Figure 10). However, pairwise comparisons showed that there was no significant difference (at the *p* < 0.05 level) in species richness between samples from Harvest 2 and Harvest 3 (*p* = 0.06).

Plants harvested at the same age also hosted bacterial communities significantly more similar to each other than any other samples (*p* = 0.0001, pseudo-*F* = 4.27), disregarding whether the plants harvested at the same time had been exposed to the exogenous soil or not. Pairwise comparisons of the three harvests showed that all age classes (3, 4, and 5 weeks old) were hosting bacterial communities significantly different from each other (*p* = 0.0001).

Examining the average bacterial community composition in samples harvested at the three different ages, results further showed that older plants hosted an increasing proportion of bacteria belonging to the orders Burkholderiales, Sphingomonadales, and Rhizobiales, while the proportion of bacteria belonging to the orders Xanthomonadales and Actinomycetales as well as the class TM7-3 decreased in abundance in older plants (Supplementary Figure S3). SIMPER analysis showed that overall, differences in the bacterial order Actinomycetales contributed most to the Bray–Curtis dissimilarities seen between plants of different ages.

##### Fungi

When comparing differences in fungal richness between harvests, we found that there was an overall significant difference between communities in plants of different ages (*p* = 0.05, *F* = 3.24; Figure 10), which was mainly driven by samples from Harvest 2 hosting significantly richer fungal communities than samples from Harvest 1 (*p* = 0.04). We also found that plants harvested at the same time hosted fungal communities compositionally more similar to each other than plants harvested at a different age (*p* = 0.0001 Pseudo-*F* = 1.87). Pairwise comparisons of the different harvests showed that they were all significantly different from each other, but that the plants harvested furthest apart in age (Harvest 1 and Harvest 3) were the most dissimilar (*p* = 0.0001).

Examining the compositional differences between harvests showed that the fungal community was heavily dominated by the genus *Fusarium*, which, on average, made up 75.6% of the community in samples across all treatments and harvests (Supplementary Figure S4).

## Discussion

### Is There a Window of Opportunity for Microbial Colonization in the Root Microbiota?

Plants were able to take up new microbial associations throughout their lifecycle; thus, there does not seem to be a discrete “window of opportunity” for plants to acquire root microbial associations. In our first experiment, soil exposure led to increased bacterial species richness, but the effect on fungal communities was less pronounced. While our study documented community composition in the roots before and after inoculation, further studies, using mock communities with known quantities of traceable fungi and bacteria as an inoculum, would provide additional information on how the assemblage dynamics affect future community composition in the root microbiota.

### Does Timing of Soil Exposure Affect Community Development in the Root Microbiota?

The timing of soil exposure was important for shaping the composition of root microbiota, as plants exposed to the soil inoculum at different developmental stages hosted distinct bacterial and fungal communities at maturity. It has previously been argued that the developmental stage of the plant host asserts strong selective forces on the composition of its root microbial community (Mougel et al., 2006; Houlden et al., 2008; Yu et al., 2012; Chaparro et al., 2013, 2014). Conversely, it has also been argued that abiotic factors (such as soil type, soil chemistry, and climate) could determine which microbes persist in the root microbiota (Buyer et al., 1999; Heuer et al., 2002; Singh et al., 2007), suggesting that plants exposed to the same soil conditions for the same amount of time would host similar microbial communities. Results here show that neither of these theories alone explain the variation in root microbiota among individual plants as not all plants of the same age hosted equivalent microbial communities, and not all plants exposed to the soil inoculum for the same amount of time hosted communities with similar composition. Instead, soil and plant factors are likely both affecting community assembly in the root microbiota (Marschner et al., 2001; Garbeva et al., 2008; Berg and Smalla, 2009; Lundberg et al., 2012; Zhao et al., 2019; Yu et al., 2021).

In our study, plants inoculated at the seed stage formed distinct communities of bacteria and fungi compared to plants inoculated at later developmental stages. Previous studies have presented contradicting ideas of the relative importance of the seed microbiome vs. the microbial communities of the soil in which the plant germinates (Buyer et al., 1999; Nelson, 2004). It has been argued that the seed microbiome constitutes a potential reservoir for root-colonizing microbes that gives them early access to colonizing the developing root system (Barret et al., 2014), while others have claimed that once a seed enters the soil, the root microbiota will mainly be recruited from the bulk soil (Normander and Prosser, 2000; Green et al., 2006).

Based on our findings, plants inoculated with microbes provided through a soil slurry at the developmental stage of seed were more receptive to microbial colonization from the soil inoculum, while older plants retained a more similar community composition to un-inoculated plants. This suggests that microbial exposure during seed germination may have lasting effects on the root microbiota, creating variation among plants of the same age.

### Does Exposure to Novel Soil Communities Affect Community Development in the Root Microbiota?

Plant exposure to novel communities was important for shaping bacteria root microbiota only for 1-week-old seedlings. In comparison, plants exposed to novel microbes as seeds, or after 2 weeks of germination were resistant to colonization by novel microbes and hosted bacterial and fungal communities more similar to those of plants inoculated only with resident soil microbes. Previous studies have found that root communities might not be equally stable across plant age classes when examining fungal pathogen establishment (Hart and Endo, 1981; Raftoyannis and Dick, 2002), and application of beneficial rhizobacteria (Bashan, 1986), but their results showed that younger plants are more susceptible to colonization by introduced microbes.

### Differences Between Responses in Bacterial and Fungal Communities

Overall, our results confirmed the prediction that bacterial and fungal communities would differ in their responses, as fungal communities were more resistant to perturbation than bacterial communities. The different responses to perturbation in bacteria and fungi could be due to differences in dispersal and colonization strategies between microbes. In our study, the majority of bacteria would be easily dispersed into the system through the addition of the exogenous soil inoculum, whereas fungal dispersal may have been hindered because mycelial growth from already established hyphal networks was not facilitated. This may have made it easier for bacteria than fungi to disperse into the system and colonize roots. In addition, while bacteria have been shown to effectively colonize root systems within 24 h of soil introduction (Edwards et al., 2015), fungi generally require more time to germinate from spores and extend hyphae in order to colonize roots (Ishida et al., 2008); therefore, it is possible that 3 weeks might not have been enough time to detect a measurable response to perturbation.

Another difference between bacteria and fungi was that fungal species richness varied little across all plants, compared to bacterial communities. This may be because, at an early stage, fungal communities were dominated by a single fungal genus, *Fusarium*, which then retained dominance in the roots throughout all of the different ages and perturbation timings examined. It has previously been documented that root colonization by mycorrhizal fungi (Kennedy et al., 2009; Werner and Kiers, 2015) and dominance in fungal wood decomposer communities (Fukami et al., 2010) is affected by historical events, with early colonizers gaining an advantage in colonizing root surfaces. In contrast, bacterial communities showed clear fluctuations in community composition, both as a response to perturbation and in association with the age at which plants were harvested. These results reinforce the idea of bacterial taxa being more opportunistic and transient members of the root microbiota, as well as the importance of studying a broad set of microbial groups to get a holistic view of dynamics in the root microbiota, and the factors that affect its composition.

## Application and Future Directions

This work provides unique insight into how the timing of soil exposure could impact root bacterial and fungal community dynamics throughout the life of the plant. Overall, our main conclusions were that plants of all ages were able to take up new microbial associations when introduced to the soil. The timing of introduction to soil created distinct variation in root microbiota between mature plants harvested at the same age.

These studies support the idea that there are stages in plant development when plants are more receptive to colonization by microbial communities. Understanding how microbial communities are formed, and the extent to which they can be shaped and manipulated, could be of great importance in managed ecosystems. For example, because stability in the root microbiota fluctuated during early development, manipulation of root microbiota composition and structure might be easier to achieve whether treatments are applied after seed germination but before significant root development. However, it also suggests that 1-week-old plants might be extra sensitive to pathogen establishment and disturbances in the root microbiota.

Our findings contribute to the idea that events during a plant’s life, such as soil perturbation, have the potential to increase individual variation in root microbiota within a plant community by altering the direction of community development.

## Data Availability Statement

The original contributions presented in the study are publicly available. This data can be found at: https://figshare.com/articles/dataset//1420638.

## Author Contributions

KA designed, executed, analyzed, and wrote the manuscript. DR helped to write the manuscript and created figures. BP helped with project design, analyses, and writing. MH designed and helped to write the manuscript. All authors contributed to the article and approved the submitted version.

## Funding

MH was funded by NSERC Discovery.

## Conflict of Interest

The authors declare that the research was conducted in the absence of any commercial or financial relationships that could be construed as a potential conflict of interest.

## Publisher’s Note

All claims expressed in this article are solely those of the authors and do not necessarily represent those of their affiliated organizations, or those of the publisher, the editors and the reviewers. Any product that may be evaluated in this article, or claim that may be made by its manufacturer, is not guaranteed or endorsed by the publisher.
