# Supplementary material for: Community Assembly and Stability in the Root Microbiota During Early Plant Development
Source: Front Microbiol. 2022 Apr 21;13:826521. doi: 10.3389/fmicb.2022.826521 (PMC9069014; doi:10.3389/fmicb.2022.826521)
Supplement: Supplementary file 1 [file Data_Sheet_1.PDF]

Table S1. Window of opportunity. Number of samples with successful sequence amplification available for analysis from each treatment and harvest, comparing the rarefied dataset (bacteria: 2000 sequences/sample, fungi: 1000 sequences/sample) with the non-rarefied dataset.

Rarefied dataset

	**Harvest 1**		**Harvest 2**		**Harvest 3**	
**Age at inoculation**	*Bacteria*	*Fungi*	*Bacteria*	*Fungi*	*Bacteria*	*Fungi*
**9 weeks**	4	5	4	5	3	3
**8 weeks**	4	2	5	5	4	4
**7 weeks**	5	3	5	2	5	5
**1 week**	1	1	2	0	2	1
**0 weeks**	Not available	Not available	3	0	5	3

Non-rarefied dataset

	**Harvest 1**		**Harvest 2**		**Harvest 3**	
**Age at inoculation**	*Bacteria*	*Fungi*	*Bacteria*	*Fungi*	*Bacteria*	*Fungi*
**9 weeks**	5	5	5	5	5	4
**8 weeks**	5	3	5	5	4	5
**7 weeks**	5	3	5	4	5	5
**1 week**	4	1	4	0	2	3
**0 weeks**	Not available	Not available	4	1	5	5

Table S2. Resistance hypothesis. Additional information about the origin and properties of the resident and exogenous soils used in the experiment.

	**Resident soil**	**Exogenous soil**
**Location**	Okanagan campus grounds of University of British Columbia in Kelowna, BC, Canada	Glacier national park, Montana, USA
**Coordinates**	49.939975N, -119.399264W	48.288020N, -113.205170W
**Elevation**	344m	2064m
**Ecosystem**	anthropogenic/disturbed/urban	subalpine grassland
**Plant community**	*Setaria viridis, Tragopogon dubius, Achillea millefolium, Senecio vulgaris, Chenopodium album, Kochia scoparia, Rumex crispus,*	*Dasiflora fruticosa, Festuca scabrella, Pseudoroegneria spicata, Lupinus spp., Achillea millefolium, Townsendia parryi*
**Soil type**	clay loam	silt loam
**Soil organic matter content (LOI 360°C)**	2%	6%
**Soil colour**	Gray	black

Table S3. Resistance hypothesis. Climate data for the two regions where the soils were collected. MAT = mean annual temperature (°C), MWMT = mean warmest month temperature (°C), MCMT = mean coldest month temperature (°C), TD = temperature difference between MWMT and MCMT or continentality (°C), MAP = mean annual precipitation (mm), MSP =May to September precipitation (mm), AHM = annual heat-moisture index (MAT+10)/(MAP/1000)), SHM = summer heat-moisture index ((MWMT)/(MSP/1000))

	**Resident soil (UBC Okanagan)**	**Exogenous soil (Glacier national park)**
**MAT**	9.1	1.8
**MWMT**	20.6	13.3
**MCMT**	-1.6	-7.2
**TD**	22.3	20.5
**MAP**	362	1272
**MSP**	167	414
**AHM**	52.6	9.3
**SHM**	123.7	32.1

Table S4. Window of Opportunity. PERMANOVA results generated from a rarefied and log-transformed dataset (bacteria: 2000 sequences/sample, fungi: 1000 sequences/sample) and a non-rarefied dataset that was rlog-transformed. The data compares bacterial and fungal communities from plants harvested before inoculation (Harvest 1), plants inoculated at different ages but exposed to the inocula for the same length of time (Harvest 2), and plants inoculated at different ages but harvested at the same age (Harvest 3). Results show that for bacteria, results remain significant independently of whether the data set was rarefied or rlog transformed. For fungi, the rlog transformed data set no longer showed significant differences at the α=0.05 level between communities from plants exposed to soil at different ages but harvested at the same age (Timing of inoculation, Harvest 3) as well as between plants harvested before and after inoculation (Effect of inoculation, Harvest 1 v.s. Harvest 2).

**Factor tested (in bold) and samples examined.**	**Bacteria** Rarefied (log-transformed Bray Curtis dissimilarities)		**Bacteria** (rlog-transformed Euclidian distances)		**Fungi** Rarefied (log-transformed Bray Curtis dissimilarities)		**Fungi** (rlog-transformed Euclidian distances)	
	Pseudo-F	p	Pseudo-F	p	Pseudo-F	p	Pseudo-F	p
**Timing of inoculation**								
Harvest 2	2.22	0.0002	2.26	0.0001	-	-	0.97	0.59
Harvest 3	1.45	0.008	1.29	0.02	1.38	0.03	1.17	0.06
**Effect of inoculation** Harvest 1 compared to Harvest 2	2.89	0.0001	2.13	0.0001	1.56	0.03	1.37	0.06

Table S5. Window of Opportunity. Results from running a 2-way PERMANOVA, comparing log transformed Bray Curtis distances between bacterial and fungal communities from Harvest 1 and Harvest 2 based on the factors Harvest and Age at harvest. Because of sample loss due to low amplification, we were not able to assess the interaction between harvest and age at harvest for fungal communities as several age classes were represented by too low number of samples (see table A.2. for numbers).

	**Bacteria**		**Fungi**	
	Pseudo-F	p	Pseudo-F	p
Harvest	1.61	0.04	1.16	0.24
Age at harvest	1.67	0.0001	1.17	0.09
HaXAg**	0.91	0.58	No test	No test

** Term has one or more empty cells

Table S6. Resistance hypothesis. Table showing an explanation of treatments and results of PERMANOVA analysis on log-transformed Bray-Curtis dissimilarities. Plants were either only inoculated with a resident soil (A1, B1, C1) or exposed to microbes from novel microbial communities at different ages (A2, B2, C2). In order to determine the effect of the perturbation, pair-wise comparisons were made between exposed to microbes from novel microbial communities and non-exposed to microbes from novel microbial communities samples of the same age.

Treatment name	Stage at exposition	Soil exposition	Age at harvest	Pair-wise comparison of Bray-Curtis dissimilarities (Bacteria)		Pair-wise comparison of Bray-Curtis dissimilarities (Fungi)	
				t-value	P (perm)	t-value	P (perm)
A1	Seed	Resident soil	3 weeks	1.12	0.098	1.10	0.194
A2	Seed	Exogenous soil	3 weeks				
B1	one-week-old	Resident soil	4 weeks	1.28	0.007	0.88	0.864
B2	one-week-old	Exogenous soil	4 weeks				
C1	2-weeks-old	Resident soil	5 weeks	1.02	0.347	1.05	0.288
C2	2-weeks-old	Exogenous soil	5 weeks				

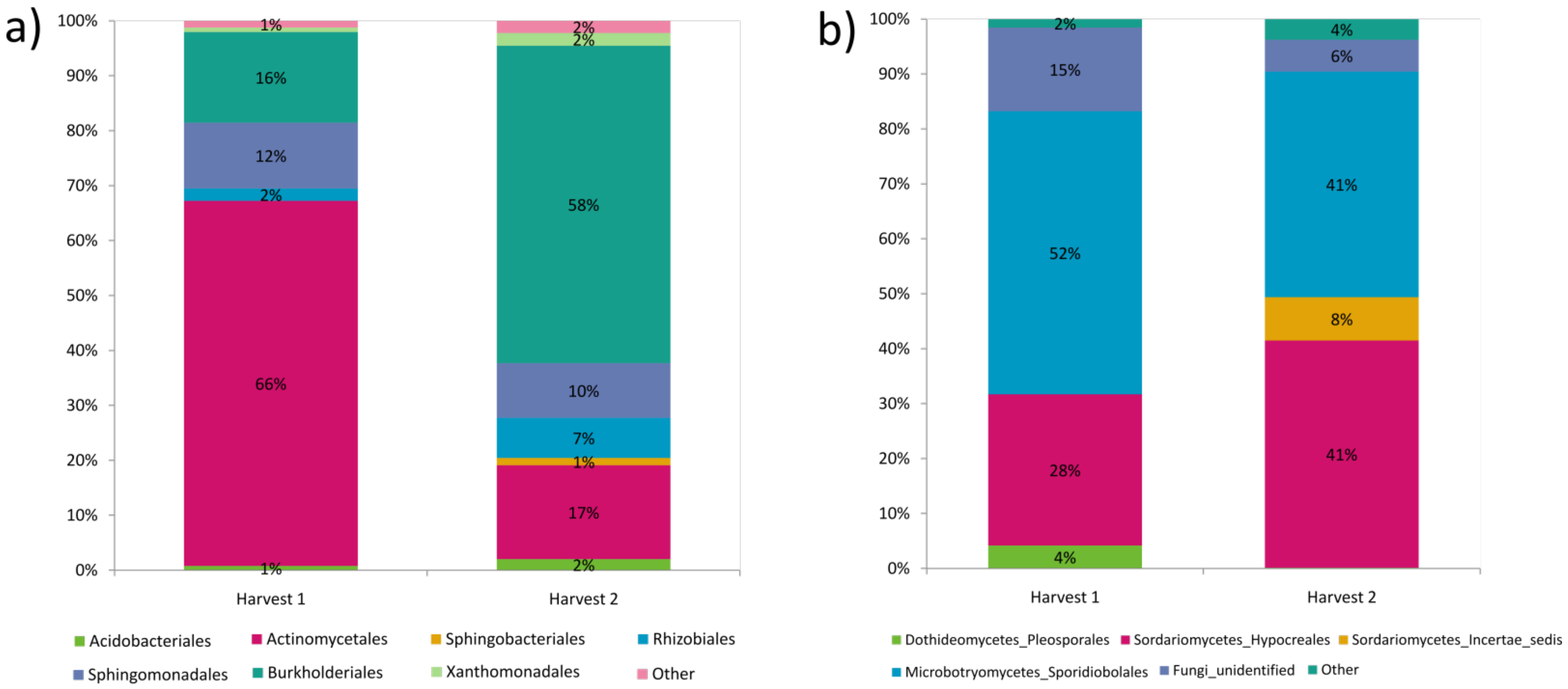

Fig. S1. Window of Opportunity. relative abundance of sequences belonging bacterial (a) and fungal (b) Orders in roots harvested at the same age (9 weeks old) prior to (Harvest 1) or two weeks after (Harvest 2) exposure to soil.

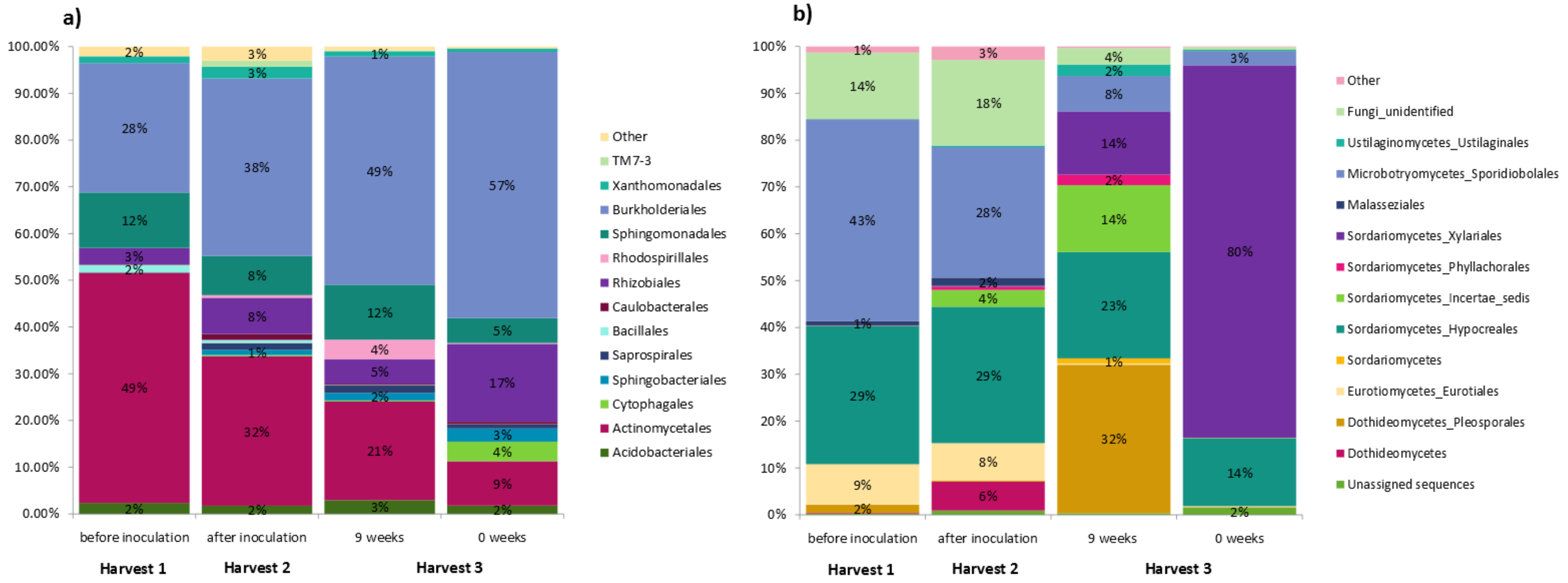

Figure S2. Average relative abundance of sequences belonging to bacterial (a) and fungal (b) orders, compared between samples harvested before (Harvest 1), after (Harvest 2), and at 12 weeks post soil exposure (Harvest 3). Orders representing less than 1% of the community have been grouped as "Other". For bacterial communities we see that Actinomycetales made up a larger portion of the average community prior to soil inoculation whereas Burkholderiales became more dominant in the root microbiota after the introduction of soil. For fungal communities, we see less prominent changes in the community after soil inoculation, but that plants inoculated from seeds form very distinct fungal communities, heavily dominated by the order Xylariales.

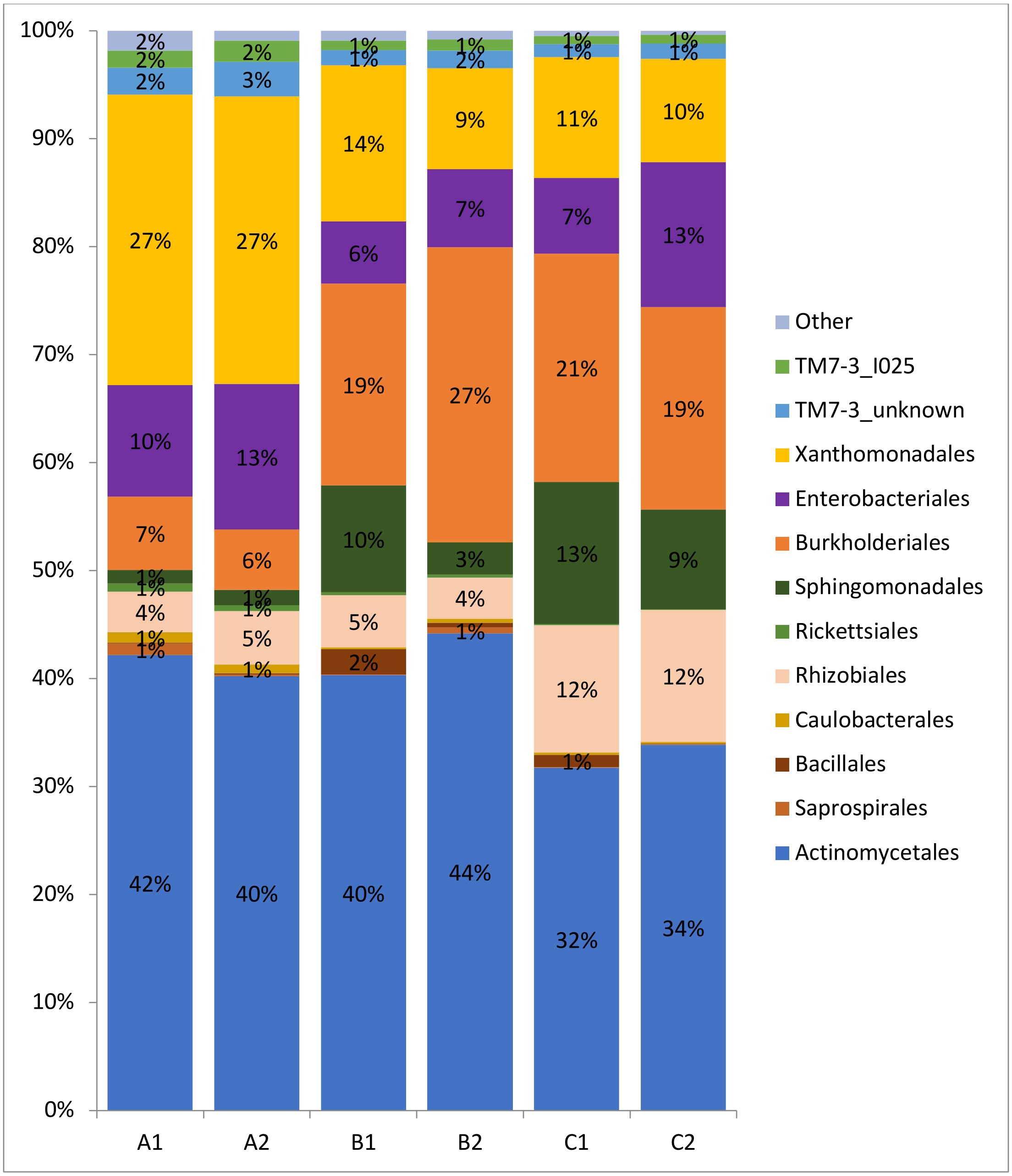

Figure S3. Comparison of the average proportion of bacterial orders found in plants from different treatments. Orders that made up less than 1% of the total community were grouped as "Other". The plants compared were perturbed either with a resident- (A1, B1, C1) or an exogenous soil (A2, B2, C2) as seeds (A), one-week-old seedlings (B) or 2-week-old seedlings (C).

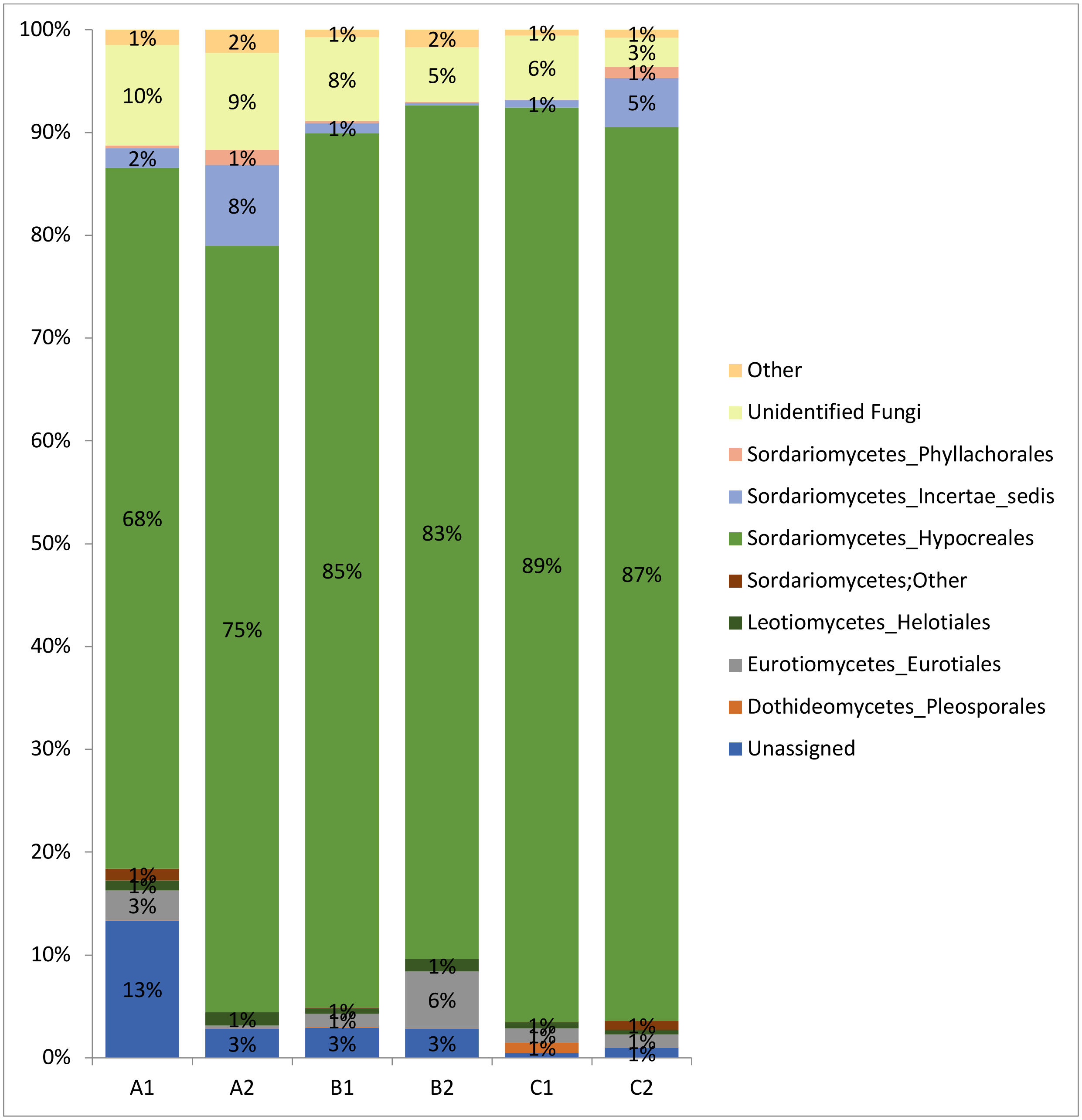

Figure S4. Average proportion of fungal orders found in plants from different treatments. Orders that made up less than 1% of the total community were grouped as "Other". The plants compared were perturbed either with a resident- (A1, B1, C1) or an exogenous soil (A2, B2, C2) as seeds (A), one-week-old seedlings (B) or 2-week-old seedlings (C).

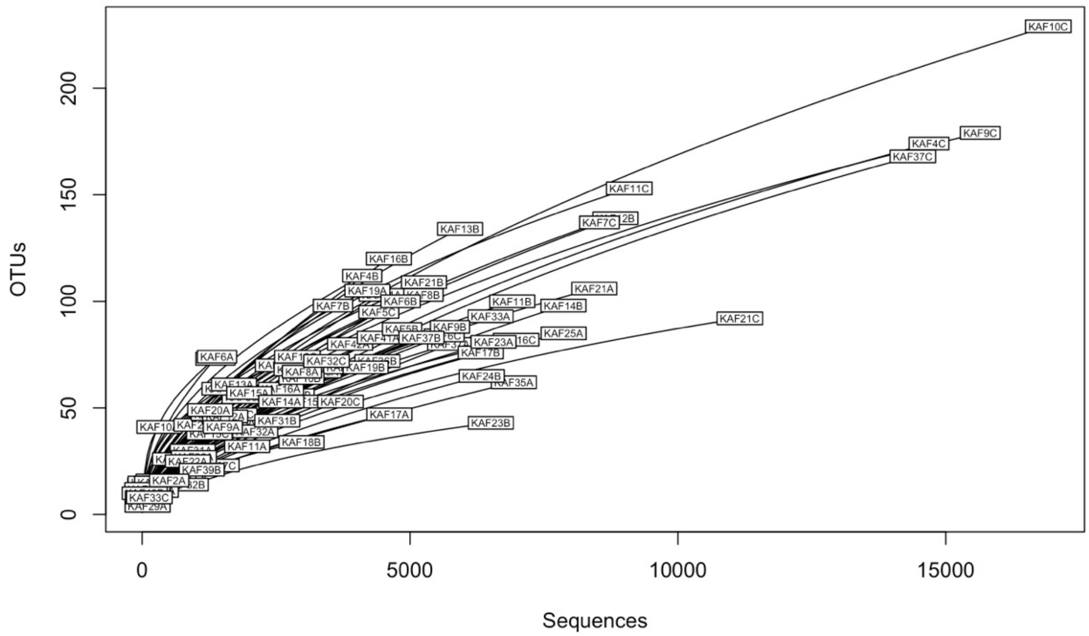

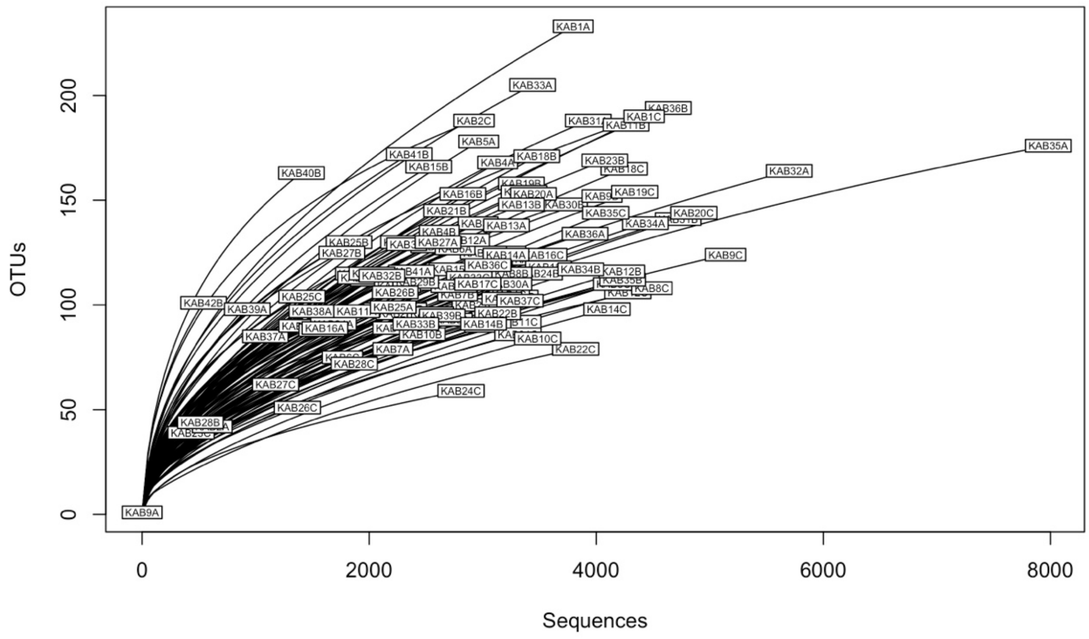

Figure S5. Rarefaction curves for bacterial (top) and fungal (bottom) sequences in the Window of Opportunity experiment.

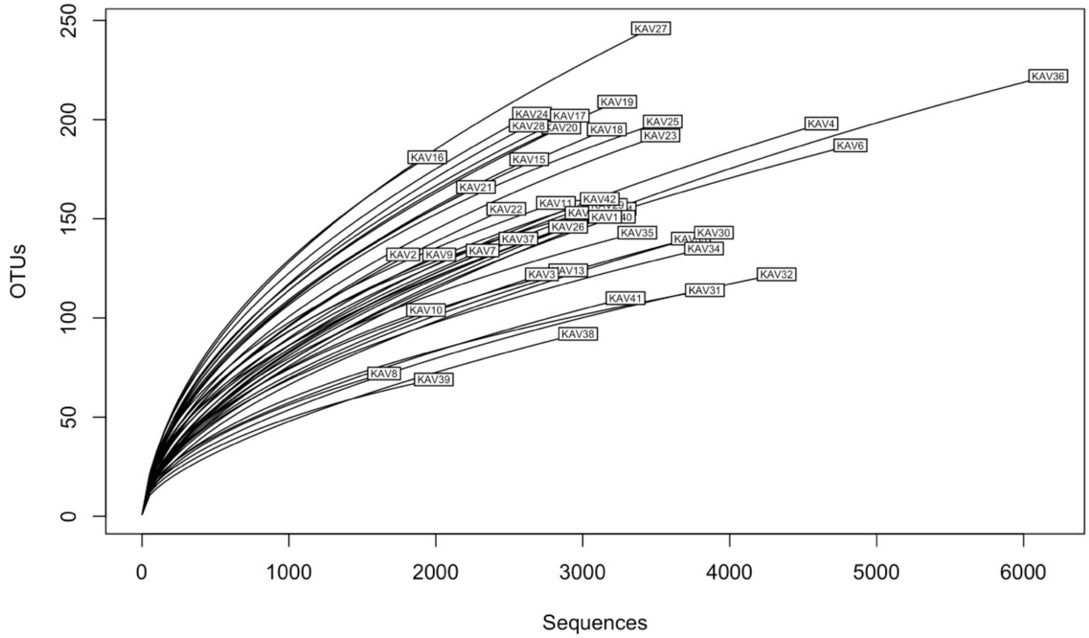

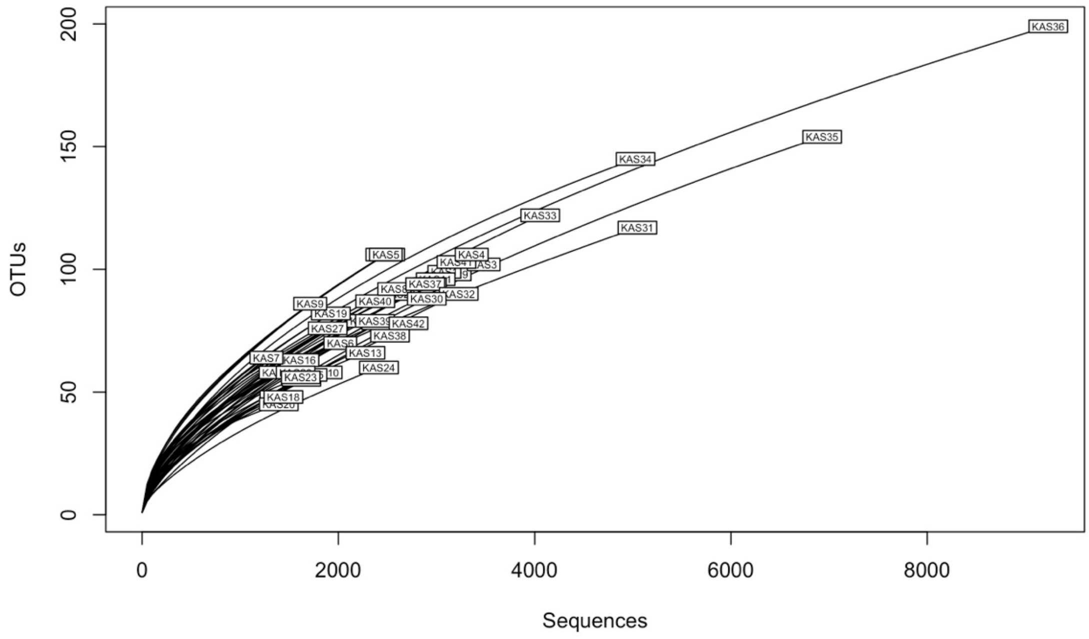

Figure S6. Rarefaction curves for bacterial (top) and fungal (bottom) sequences in the Resistence hypothesis experiment.
